# Hepcidin Protects Neuron from Hemin-Mediated Injury by Reducing Iron

**DOI:** 10.3389/fphys.2017.00332

**Published:** 2017-05-23

**Authors:** Yu-Fu Zhou, Chao Zhang, Guang Yang, Zhong-Ming Qian, Meng-Wan Zhang, Juan Ma, Fa-Li Zhang, Ya Ke

**Affiliations:** ^1^Laboratory of Neuropharmacology, School of Pharmacy, Fudan UniversityShanghai, China; ^2^Faculty of Medicine, School of Biomedical Sciences, The Chinese University of Hong KongShatin, Hong Kong

**Keywords:** hemin, intracerebral hemorrhage (ICH), neurons, iron regulatory hormone hepcidin, apoptotic cell, ferritin

## Abstract

Hemin plays a key role in mediating secondary neuronal injury after intracerebral hemorrhage (ICH) and the cell toxicity of hemin is thought to be due to iron that is liberated when hemin is degraded. In a recent study, we demonstrated the iron regulatory hormone hepcidin reduces brain iron in iron-overloaded rats. Therefore, we hypothesized that hepcidin might be able to reduce iron and then protect neurons from hemin or iron-mediated neurotoxicity in hemin-treated neuronal cells. Here, we tested the hypothesis and demonstrated that ad-hepcidin and hepcidin peptide both have the ability to suppress the hemin-induced increase in LDH release and apoptotic cell numbers, to reduce cell iron and ferritin contents, and to inhibit expression of transferrin receptor 1, divalent metal transporter 1, and ferroportin 1 in hemin-treated neurons. We conclude that hepcidin protects neuron from hemin-mediated injury by reducing iron via inhibition of expression of iron transport proteins.

## Introduction

Intracerebral hemorrhage (ICH) is a major public health problem (van Asch et al., 2010) and affects more than one million people worldwide annually (Liu et al., 2007; Anderson et al., 2013). ICH carries a mortality rate of 30–50% and imparts some form of disability in close to 90% of its survivors (Qureshi et al., 2009; van Asch et al., 2010). ICH occurs when a blood vessel in part of the brain becomes weak and bursts open, causing blood to leak into the brain (Qureshi et al., 2009; Sangha and Gonzales, 2011). Evidence indicates that hemin, a breakdown product of hemoglobin, plays a key role in mediating secondary neuronal injury after ICH (Qureshi et al., 2009; Babu et al., 2012; Owen et al., 2016). Hemin breakdown by the heme-oxygenase (HO) enzyme liberates extracellular free iron, which is neurotoxic by catalyzing hydroxyl radical formation and promoting oxidative stress (Wagner et al., 2003; Nagababu and Rifkind, 2004; Lou et al., 2009; Dang et al., 2011).

It has been reported that slowing iron release by inhibiting hemin breakdown could induce a decrease in cell death and reactive oxygen species (Chang et al., 2011; Chen-Roetling et al., 2014) The studies have also showed that the iron chelator including Deferoxamine Mesylate (DFO) reduces hemin and iron-mediated neurotoxicity, perihematoma edema, and neuronal damage, leading to good neurologic outcomes after ICH (Hoepken et al., 2004; Nakamura et al., 2004; Masuda et al., 2007; Belur et al., 2013; Yeatts et al., 2013). Clinical studies observe that serum ferritin (Millan et al., 2007; Pérez de la Ossa et al., 2010) or a combination of serum iron, ferritin, and transferrin (Yang et al., 2016) could predict clinical outcome in patients with ICH. These findings solidly evidenced for the critical involvement of hemin and iron in the development of brain injury after ICH.

Hepcidin is a central player in body iron homeostasis. Recent studies have revealed that this iron regulatory hormone is also widespread distributed in the brain (Zechel et al., 2006; Wang et al., 2008; Du et al., 2011; Qian et al., 2014) and has the ability to reduce brain iron in iron-overloaded rats by down-regulating iron transport proteins, playing a key role in brain iron homeostasis (Du et al., 2015; Gong et al., 2016). Therefore, hepcidin might be able to reduce hemin and iron-mediated neurotoxicity in ICH brain by reducing iron level in the brain. Under in *in vitro* conditions, this peptide might be able to protect neuronal cells from hemin and iron-mediated neurotoxicity. In the present study, we test the hypothesis and demonstrated that hepcidin has the ability to suppress the hemin-induced increase in LDH release and apoptotic cell numbers by inhibiting expression of transferrin receptor 1, and divalent metal transporter 1 and ferroportin 1, and reducing cell iron in hemin-treated neurons.

## Materials and methods

### Chemicals

Unless otherwise stated, all chemicals were obtained from Sigma Chemical Co., St. Louis, MO, USA. Mouse monoclonal anti-rat transferrin receptor 1 (TfR1) and goat anti-mouse IgG-Alexa 488 were purchased from Invitrogen, Carlsbad, CA, USA; rabbit polyclonal anti-rat divalent metal transporter 1 (DMT1, SLC11A2) and rabbit polyclonal anti-ferritin-heavy-chain (Ft-L) from Proteintech, Chicago, IL, USA; rabbit polyclonal anti-ferritin-hight-vhain (Ft-H) from Bioworld Technology Inc., Louis Park, MN, USA; and rabbit polyclonal anti-mouse ferroportin 1 (Fpn1) from Novus Biologicals, Littleton, CO, USA. Goat anti-rabbit or anti-mouse IRDye 800 CW secondary antibody was bought from Li-Cor, Lincoln, NE, USA; TRIzol reagent from Life Technologies, Carlsbad, CA, USA; and AevertAid First Strand cDNA Synthesis Kit and BCA Protein Assay Kit both from Thermo Scientific, Waltham, MA, USA. Microtubule associated protein 2 (MAP2) and astrocyte marker glial fibrillary acidic protein (GFAP) were obtained from Chemicon International, Inc. Temecula, CA, USA; the *in situ* cell death detection kit, TMR red from USA Roche, Nutley, NJ, USA. The Health Department of Hong Kong and Shanghai Government and the Animal Research Ethics Committee of The Chinese University of Hong Kong and Fudan University approved the experimental procedures of this study.

### Primary cortical neuron

Primary cortical neurons were prepared from embryonic day 16–17 Sprague-Dawley rats as previously described (Ke et al., 2006). Dissociated cortical cells were suspended in Dulbecco's modified Eagle's medium (DMEM) containing 10% fetal bovine serum (FBS; Invitrogen Life Technologies) with antibiotics (penicillin 100 U/ml, streptomycin 100 μg/ml) and then seeded on culture plates pre-coated with poly-L-lysine at a density of 1.5 × 10^6^ cells/ml. The cultures were maintained at 37°C in a humidified environment with 5% CO2 in a CO2 incubator (NAPCO 5400). After 24 h, the cultural medium was replaced with neurobasal medium supplemented with 2% B-27 and antibiotics (penicillin 100 U/ml, streptomycin 100 μg/ml). The cultured neurons were maintained in the neurobasal B27 medium for 6 days. The purity of the cultures was assessed by staining with the neuron-specific antibodies against MAP2 and the astrocyte marker GFAP. A purity of at least 98.5% was achieved.

### Construction of the hepcidin expression adenovirus (Ad-hepcidin)

Ad-hepcidin was constructed as described previously (Du et al., 2011). Hepdicin protein encoding region (Genebank NM-053469) was cloned from the rat cDNA by PCR (Du et al., 2009). The recombinated adenovirus was named “ad-hepcidin.” Propagation and purification of recombinated adenovirus were conducted by sequential centrifugation in CsCl step gradients and dialysis against 10% glycerol in phosphate-buffered saline (PBS). Finally, the virus with the titer of 2 × 10E11 plaque forming units [pfu]/μL was obtained for the infection of the brain *in vivo*. GFP-expression adenoviruses (ad-blank) were used as negative control.

### Isolation of total RNA and quantitative real-time PCR

Total RNA extraction and cDNA preparation were performed using TRIzol reagent and AevertAid First Strand cDNA Synthesis Kit in accordance with the instructions of the manufacturers, respectively. Real-time PCR was carried out using faststart universal SYBR Green master and lightcycler96. The specific pairs of primers were: hepcidin, forward primer: 5′-gaaggcaagatggcactaagca-3′; reverse primer: 5′-tctcgtctgttgccggagatag-3′ and β-actin, forward primer: 5′-gaaatcgtgcgtgacattaaagag-3′; reverse primer: 5′-gcggcagtggccatctc-3′ (Du et al., 2012). The hepcidin mRNA level of each sample was normalized to that of the β-actin mRNA. Relative mRNA level was presented as 2 [Control Ct (β-actin-Hepcidin)—Expt. Ct (β-actin-Hepcidin)]. The specific band of hepcidin was confirmed by DNA gel electrophoresis.

### Western blot analysis

The cells were washed and homogenized and then sonicated as described previously (Chang et al., 2005; Ke et al., 2005). Protein content was determined using the BCA protein Assay kit. The primary antibodies used are: mouse monoclonal anti-rat TfR1 (1:500); rabbit polyclonal anti-rat DMT1 (1:1,000), rabbit polyclonal anti-mouse Fpn1 (1:1,000), rabbit polyclonal anti-mouse Ft-L (1:1,000), and rabbit polyclonal anti-mouse Ft-H antibody (1:1,000). After being washed three times, the blots were incubated with goat anti-rabbit or anti-mouse IRDye 800 CW secondary antibody (1:5,000) for 1-h at room temperature. The intensity of the specific bands was detected and analyzed by Odyssey infrared image system (Li-Cor, Lincoln, NE, USA). To ensure even loading of the samples, the same membrane was probed with mouse monoclonal anti-actin antibody at a 1:2,000 dilution.

### Tunel assay

Terminal deoxynucleotidyl transferase-mediated biotinylated-dUTP nick-end labeling (TUNEL) staining was performed by use of the *in situ* cell death detection kit, TMR red according to the manufacturer's instruction. Tissue sections were analyzed with fluorescence microscopy (Zhao et al., 2008; Wu et al., 2011) and positive cells counting were done with Image pro plus 6.0 software.

### Lactate dehydrogenase measurements

Cell injury was quantitatively assessed by the measurement of LDH released from damaged cells. The quantity of LDH release in the medium was determined by the decrease in absorbance at 340 nm for NADH disappearance within 3 min (He et al., 2008; Du et al., 2010). Briefly, 500 μl of supernatant was collected from each well and mixed with 1.3 ml of NADH (0.217 mmol/l) and 1.3 ml of sodium pyruvate (1.77 mmol/l) in the modiWed Krebs–Henseleit buffer (118 mmol/l NaCl, 4.8 mmol/l KCl, 1 mmol/lKH_2_PO_4_, 24 mmol/l NaHCO_3_, 3 mmol/l CaCl_2_, 0.8 mmol/l MgPO_4_, pH 7.4) for 30 s at 37°C. The activity was spectrophotometrically measured by the use of an ELX-800 microplate assay reader (Elx800, Bio-tek, USA).

### Measurement of cell iron

Cell iron was determined using a graphite furnace atomic absorption spectrophotometer (GFAAS, Perkin-Elmer, Analyst 100) as described previously (Zhu et al., 2016). Cells were diluted with HEPES buffer and homogenized with a sonicator (MSE Soniprep 150 Ultrasonic Disintegrator, MSE Scientific Instruments, England). A 50-μl portion of the homogenate was added to an equal volume of ultra-pure nitric acid in a 0.5 ml polypropylene microfuge tube, digested for 48 h at 50°C, and diluted with 3.12 mmol/L nitric acid for iron analysis. Standard curves (0–40 ppb) were prepared by diluting iron standard with blanks prepared from homogenization reagents in 0.2% HNO3. Standards and digested samples were read in triplicate by injecting 50 μl aliquots including 0.05 mg Mg(NO3)2 as matrix modification into graphite furnace. Absorbance readings at 248.3 nm, slit at 0.2 nm, pretreatment temperature at 1400°C, atomization temperature at 2400°C were recorded.

### Statistical analysis

Statistical analyses were performed using SPSS software for Windows (version 15.0; SPSS, Inc., Chicago, IL). Data were presented as mean ± SEM. The difference between or among the means was determined by Kruskall-Wallis test followed by Mann-Whitney test for multiple comparisons or One-Way or Two-way ANOVA in appropriate experiments followed by Newman-Keuls *post*-*hoc* test. A probability value of *P* < 0.05 was taken to be statistically significant.

## Results

### Ad-hepcidin increased hepcidin expression in primary cortical neuron of rats

**Figure 1:**
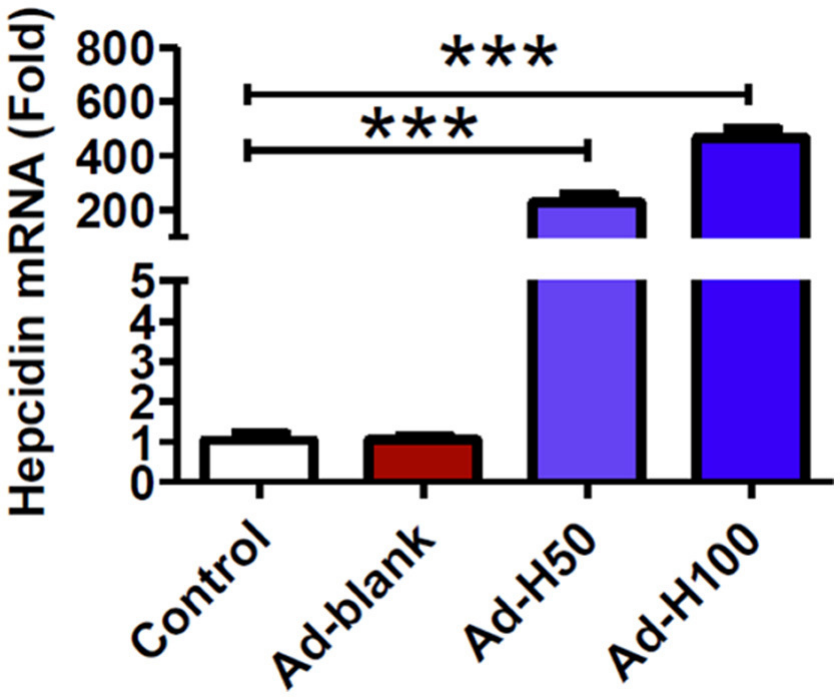
**Ad-hepcidin increased hepcidin expression in cultured cortical neurons**. The cultured cortical neurons were incubated with 0, 50, or 100 MOI of ad-hepcidin or ad-blank for 40-h and expression of hepcidin mRNA was then determined by quantitative real-time PCR as described in Section “Methods and Materials.” Results are presented as mean ± SEM (*n* = 5). ^***^P < 0.001 vs. Control.

We first examined the effects of treatment with ad-hepcidin on the expression of hepcidin mRNA in primary cortical neuron. It was found that infection with ad-hepcidin induced a significant increase in the expression of hepcidin mRNA in cultured neurons (Figure 1). The levels of hepcidin mRNA in neuronal cells treated with 50 or 100 MOI of ad-hepcidin both were significantly higher than those in control cells, being 226.5 ± 28.6-fold (MOI 50) and 466.6 ± 32.13-fold (MOI 100) of the control values; respectively. Infection of the cells with ad-blank had no effect on the expression of hepcidin mRNA, no difference being found between the neurons in Ad-Blank and control groups (Figure 1).

### Ad-hepcidin and hepcidin peptide suppressed the hemin-induced increase in LDH release in cultured neuron

**Figure 2:**
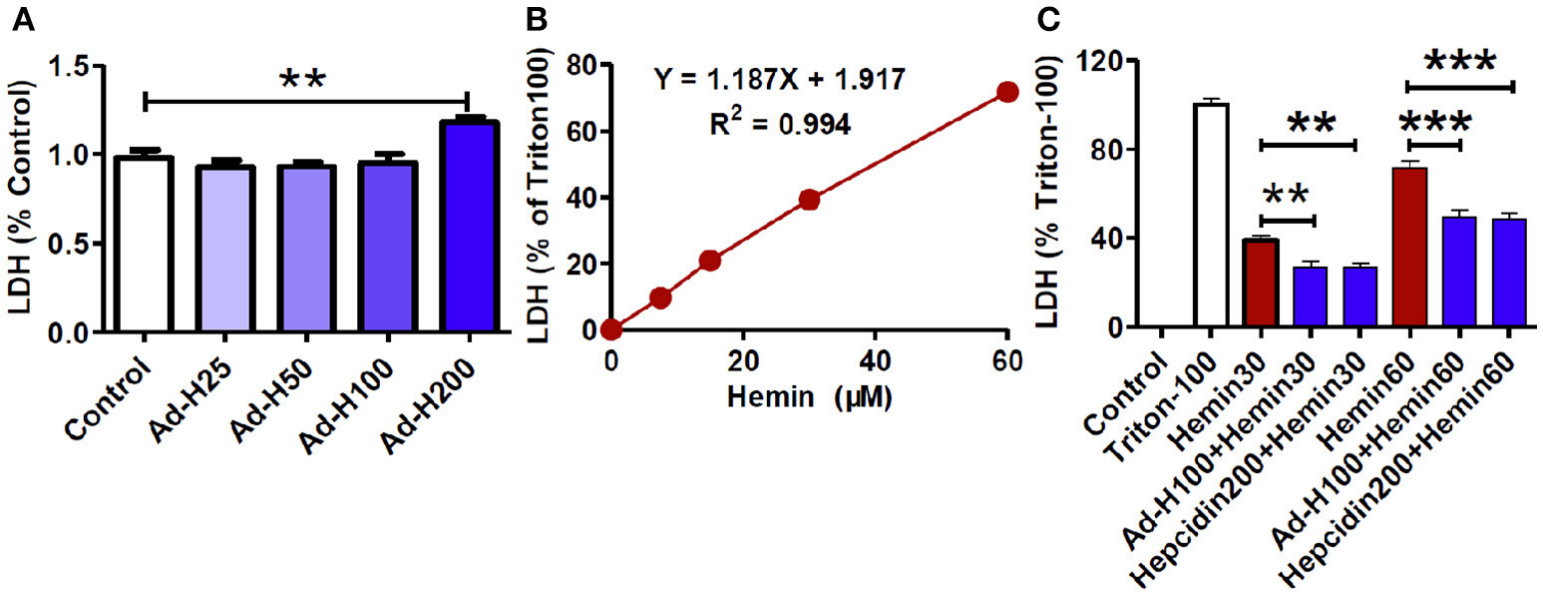
**Ad-hepcidin and hepcidin peptide suppressed the hemin-induced increase in LDH release in cultured neuron**. The cultured neurons were treated with 0, 25, 50, 100, and 200 MOI of ad-hepcidin for 40-h **(A)**; 0, 7.5, 15, 30, and 60 μM of hemin for 24-h **(B)**; or 30 (Hemin30), or 60 μM (Hemin60) of hemin for 24-h, or 100 MOI of ad-hepcidin for 16-h and then 30 (Ad-H100+Hemin30), or 60 μM (Ad-H100+Hemin60) of hemin for 24-h, or co-incubated with 200 nM of hepcidin peptide with 30 μM (Hepcidin200+Hemin30) or 60 μM (Hepcidin200+Hemin30) of hemin for 24-h **(C)**; respectively. The contents of LDH released from neurons were then measured as described in Section “Methods and Materials.” Results are presented as mean ± SEM (*n* = 5). ^**^P < 0.01, ^***^P < 0.001 vs. Control **(A,B)** or Hemin 30 **(C)**.

We then investigated the effects of different concentrations of ad-hepcidin on neuronal integrity or viability by incubating cultured neurons with 0, 25, 50, 100, and 200 MOI of ad-hepcidin for 40-h respectively and then examining the contents of LDH release from neurons. Treatment with 200 MOI of ad-hepcidin induced a significant increase in LDH release from neurons (Figure 2A). The contents of LDH release from control neurons were significantly lower than those from the neurons treated with 200 MOI of ad-hepcidin, but not different from those from neurons treated with 25, 50, and 100 MOI of ad-hepcidin. This indicated that ad-hepcidin at the lower concentrations (i.e., 50–100 MOI) had no marked-effects on neuronal integrity or viability, and 100 MOI of ad-hepcidin was hence chosen to be used in the following experiments.

We also observed the effects of different concentrations of hemin on neuronal integrity or viability by incubating the cultured neurons with 0, 7.5, 15, 30, and 60 μM of hemin for 24-h respectively and then examining the contents of LDH release from neurons. It was found that the contents of LDH release from neurons progressively increased with the concentrations of hemin added (Figure 2B). The amounts of LDH release from neurons treated with 30 and 60 μM of hemin were 39.30 ± 1.76 and 71.77 ± 2.07%; respectively, implying that these concentrations of hemin could induce neuron injury under our experimental conditions.

To find out whether hepcidin was able to protect neurons from hemin-induced damage, the cells were treated with ad-hepcidin (100 MOI), or ad-blank for 16-h and then with 30 or 60 μM of hemin for 24-h or incubated with hepcidin peptide (200 nM) plus 30 or 60 μM of hemin for 24-h. The contents of LDH released from neurons in groups of Ad-hepcidin100 + Hemin30 (27.07 ± 2.25%) and Hepcidin200 + Hemin30 (26.63 ± 1.96%) or Ad-hepcidin100 + Hemin60 (49.21 ± 3.25%) and Hepcidin200 + Hemin60 (48.17 ± 2.97%) were significantly lower than those from neurons in groups of Hemin30 (38.66 ± 2.11%) or Hemin60 (71.76 ± 2.22%); respectively (Figure 2C). The findings indicated that ad-hepcidin and hepcidin peptide both had a significant role to protect neurons from hemin-induced damage.

### Hepcidin protected neurons from hemin-induced apoptosis

**Figure 3:**
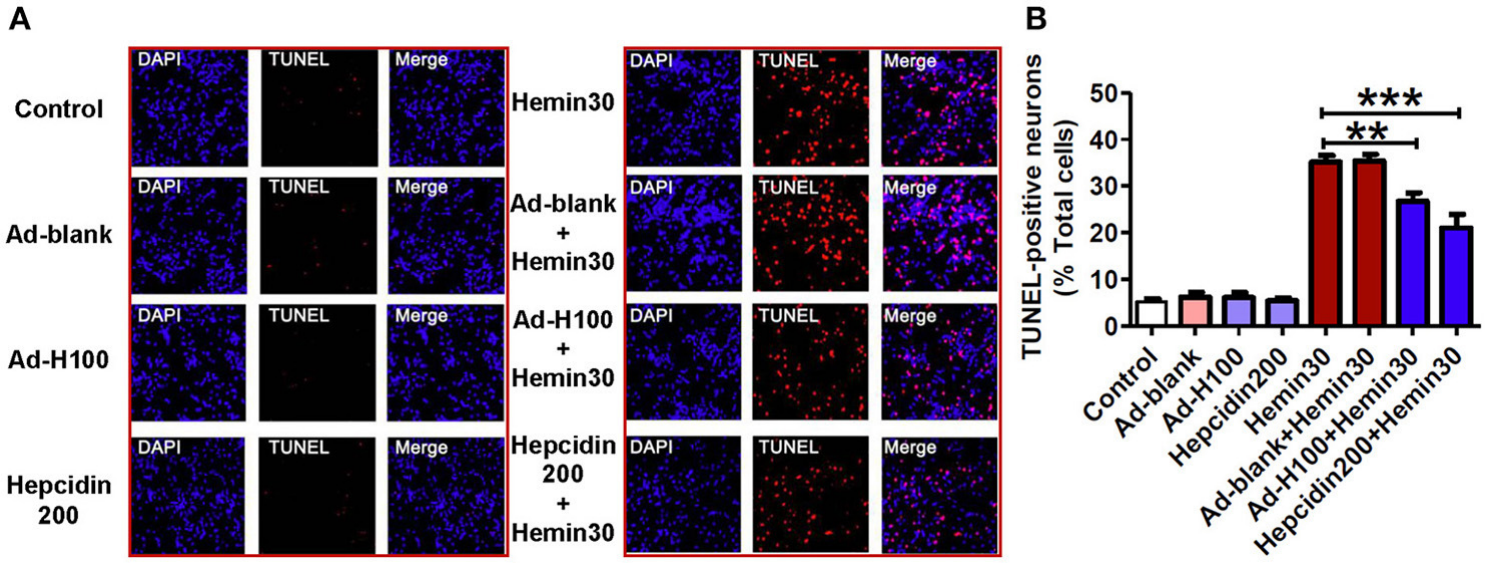
**Hepcidin protected neurons from hemin-induced apoptosis**. The cultured neurons were treated with 0 (Control) or 100 MOI of ad-hepcidin (Ad-H100), ad-blank (Ad-blank) for 40-h, 200 nM of hepcidin peptide (Hepcidin200) or 30 μM of hemin (Hemin30) for 24-h; or 100 MOI of ad-hepcidin or ad-blank for 16-h and then with 30 μM of hemin for 24-h (Ad-H100+Hemin30 or Ad-blank+Hemin30) or co-incubated with 200 nM of hepcidin peptide and 30 μM of hemin (Hepcidin200+Hemin30) for 24-h; respectively. The numbers of apoptotic cells (% total cells) were assessed by TUNEL staining analysis as described in Section “Methods and Materials.” **(A)**: A representative experiment of TUNEL staining analysis, **(B)**: TUNEL-positive neurons (% total cells). Results are presented as mean ± SEM (*n* = 5). ^**^P < 0.01, ^***^P < 0.001 vs. Hemin 30.

To further confirm the neuro-protective role of hepcidin, we then assessed effects of ad-hepcidin or hepcidin peptide on hemin-induced apoptosis by TUNEL staining analysis. The neurons were treated with ad-hepcidin (100 MOI) or ad-blank for 16-h and then with 30 μM of hemin for 24-h or incubated with hepcidin peptide (200 nM) plus 30 μM of hemin for 24-h. The findings showed that very few TUNEL-positive cells could be detected in the control, and also in ad-blank, ad-hepcidin and hepcidin peptide groups (Figure 3). However, treatment with hemin or hemin plus ad-blank was found to induce a significant increase in the numbers of TUNEL-positive cells or apoptotic cells (% of total cells). The numbers of TUNEL-positive cells were markedly higher in Hemin and Hemin+ad-blank groups than those in the controls, and also in Hemin+ad-hepcidin and Hemin+hepcidin peptide groups. This demonstrated that pre-treatment with ad-hepcidin or hepcidin peptide could significantly suppress the hemin-induced increase in the numbers of apoptotic cells and reveled that ad-hepcidin or hepcidin peptide have the ability to protect neurons from hemin-induced apoptosis.

### Hepcidin decreased iron and ferritin contents in neurons treated with hemin

**Figure 4:**
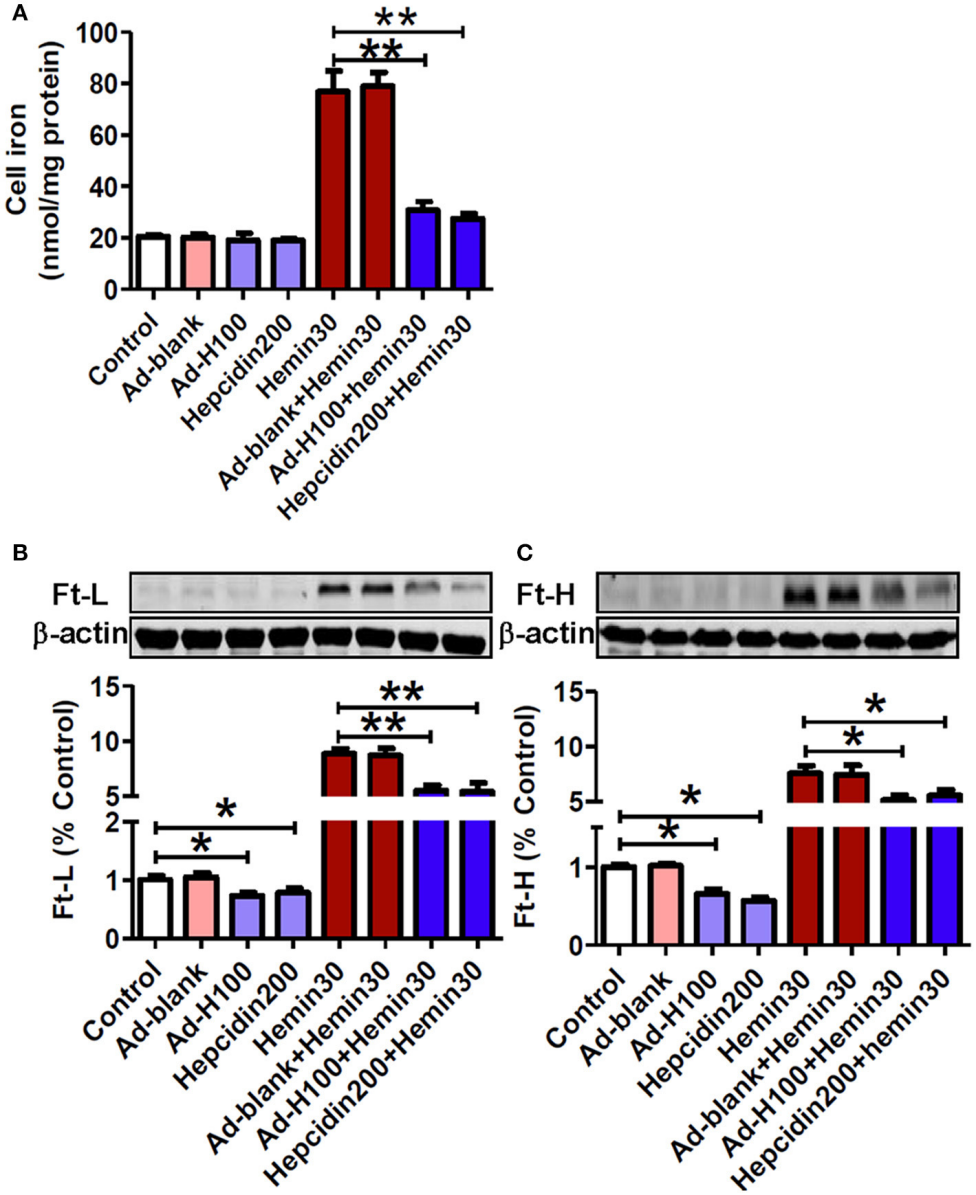
**Hepcidin decreased iron and ferritin contents in neurons treated with hemin**. The cultured neurons were treated with 0 (Control) or 100 MOI of ad-hepcidin (Ad-H100), ad-blank (Ad-blank) for 40-h, 200 nM of hepcidin peptide (Hepcidin200) or 30 μM of hemin (Hemin30) for 24-h; or 100 MOI of ad-hepcidin or ad-blank for 16-h and then with 30 μM of hemin for 24-h (Ad-H100+Hemin30 or Ad-blank+Hemin30) or co-incubated with 200 nM of hepcidin peptide and 30 μM of hemin (Hepcidin200+Hemin30) for 24-h; respectively. Cell iron contents **(A)** and ferritin-light-chain (Ft-L) **(B)** and ferritin height chain (Ft-H) **(C)** were, respectively, determined by using a graphite furnace atomic absorption spectrophotometer or western blot analysis as described in Section “Methods and Materials.” Results are presented as mean ± SEM (*n* = 5). ^*^P < 0.05, ^**^P < 0.01 vs. Control or Hemin 30.

In order to answer to the question of why hepcidin could protect neurons from hemin-induced injury and apoptosis, we investigated the effects of ad-hepcidin and hepcidin peptide on cell iron contents and ferritin levels in neurons treated with hemin. Iron was investigated here because the cell toxicity of hemin is thought to be due to iron that is liberated when hemin is degraded (Owen et al., 2016). No differences in iron contents were found among the neurons in the groups of control, ad-blank, ad-hepcidin, and hepcidin peptide. However, iron contents in neurons treated with hemin or hemin plus ad-blank were showed to be significantly higher than those in the controls, while iron contents in neurons treated with ad-hepcidin or hepcidin peptide plus hemin were markedly lower than those in neurons treated with hemin only (Figure 4A). This evidenced a inhibiting role of hepcidin on the hemin-induced increase in cell iron.

Western blot analysis showed that there were no differences in Ft-L (Figure 4B) and Ft-H (Figure 4C) contents between the control and ad-blank-treated neurons. The same as its effect on cell iron, treatment with hemin only also led to a significant increase in both Ft-L and Ft-H contents in neuronal cells, while pre-treatment with ad-hepcidin and co-incubation with hepcidin peptide both suppressed the expression of Ft-L and Ft-H proteins in neurons no matter treated with or without hemin. There were no differences in Ft-L and Ft-H contents between neuronal cells treated with hemin and hemin plus ad-blank.

### Hepcidin inhibited expression of transferrin receptor 1, and divalent metal transporter 1 and ferroportin 1 in hemin-treated neurons

**Figure 5:**
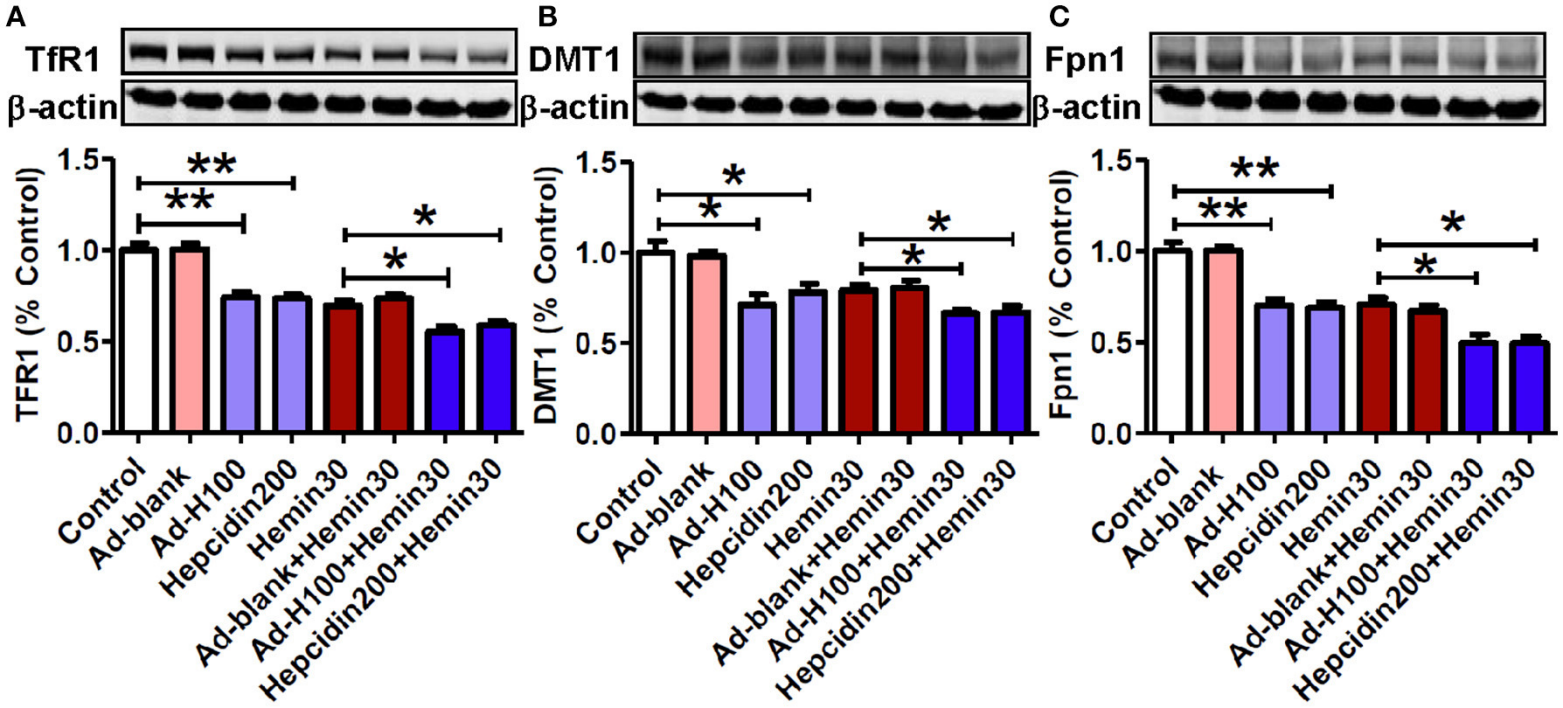
**Hepcidin inhibited expression of transferrin receptor 1, divalent metal transporter 1, and ferroportin 1 in hemin-treated neurons**. The cultured neurons were treated with 0 (Control) or 100 MOI of ad-hepcidin (Ad-H100), ad-blank (Ad-blank) for 40-h, 200 nM of hepcidin peptide (Hepcidin200) or 30 μM of hemin (Hemin30) for 24-h; or 100 MOI of ad-hepcidin or ad-blank for 16-h and then with 30 μM of hemin for 24-h (Ad-H100+Hemin30 or Ad-blank+Hemin30) or co-incubated with 200 nM of hepcidin peptide and 30 μM of hemin (Hepcidin200+Hemin30) for 24-h; respectively. The expression of TfR1 **(A)**, DMT1 **(B)**, and Fpn1 **(C)** proteins was determined by western blot analysis as described in Section “Methods and Materials.” Results are presented as mean ± SEM (*n* = 5). ^*^P < 0.05, ^**^P < 0.01 vs. Control or Hemin 30.

In order to find out the mechanisms by which hepcidin restores the hemin-induced changes in cell iron contents, we further examined effects of hepcidin on the expression of two major iron importers TfR1 and DMT1 and the only-known iron exporter Fpn1. In contrast to its effect on cell iron and ferritin contents, treatment with hemin resulted in a remarkable decrease not only in iron importers TfR1 (Figure 5A) and DMT1 (Figure 5B) but also iron exporter Fpn1 (Figure 5C). And pre-treatment with ad-hepcidin and co-incubation with hepcidin peptide both suppressed the expression of all three proteins in neurons treated with hemin as well as without hemin. There were no differences in expression of TfR1, DMT1, and Fpn1 between the control and ad-blank-treated or hemin-treated and hemin+ ad-blank-treated neurons.

## Discussion

Hemin, the oxidative form of heme, is a clinically relevant hemoglobin oxidation product that is present at high micromolar concentrations in intracranial hematomas (Kajikawa et al., 1979; Regan et al., 2004). Growing evidence suggests that this highly reactive compound is potentially cytotoxic and is responsible for much of the secondary damage following ICH (Babu et al., 2012; Kwon et al., 2013). Its mechanism of action is thought to occur through oxidative stress and the activation of caspases and then apoptotic process, resulting in the injury of brain cells including neurons (Regan et al., 2001; Wang et al., 2002; Goldstein et al., 2003).

In the present study, we showed that continuous exposure of the cultured neurons to 7.5–60 μM of hemin for 24-h produced concentration-dependent neuronal death, as detected by LDH release, and 30 μM of hemin for 24-h induced a significant increase in the numbers of apoptotic cells, as evidenced by TUNEL staining analysis. We also demonstrated for the first time that ad-hepcidin and hepcidin peptide both could significantly suppress the hemin-induced increase in LDH release and the numbers of apoptotic cells in this primary cell culture model of hemin toxicity. These findings provided evidence that hepcidin had a significant role to protect neurons from hemin-induced injury by maintaining neuronal integrity and inhibiting hemin-induced apoptosis.

The cell toxicity of hemin is thought to be due to iron that is liberated when hemin is degraded by heme oxygenases (Owen et al., 2016). Iron levels due to hemin degradation may increase up to 3-fold and remain elevated for 1 month, causing continued brain injury after ICH (Wagner et al., 2003; Wu et al., 2003). These data prompted us to consider whether neuro-protective role of hepcidin might be mediated its role to reduce iron in hemin-treated neurons as it did in iron-over-loaded brain (Du et al., 2015). Therefore, we investigated the effects of ad-hepcidin and hepcidin peptide on cell iron contents and ferritin levels in neurons treated with hemin. We demonstrated that the increase in iron contents and ferritin expression, induced by treatment with hemin, could be significantly attenuated by pre-treatment with ad-hepcidin or co-incubation with hepcidin peptide. The findings provided solid evidence for our hypothesis and confirmed that hepcidin protect neuron from hemin-mediated injury at least partly by reducing iron in the cultured neurons.

To answer the question of how hepcidin reduces iron contents and ferritin expression in neuronal cells, we further examined effects of hepcidin on the expression of two major iron importers TfR1 and DMT1 and the only-known iron exporter Fpn1. The proteins TfR1, DMT1 were examined because they are two major iron uptake proteins, while Fpn1 was investigated because it is the only known iron exporter, and the expression of these three proteins played a central role in cell iron balance. It was found that pre-treatment with ad-hepcidin or co-incubation with hepcidin peptide was able to significantly reduce expression of TfR1, DMT1, and Fpn1 in neurons treated with hemin, while ad-blank had no such a effect. The findings plus the results of our recent study (Du et al., 2015; Gong et al., 2016) supported that hepcidin reduces cell iron by down-regulating iron uptake and release proteins in the cultured neurons.

**Figure 6:**
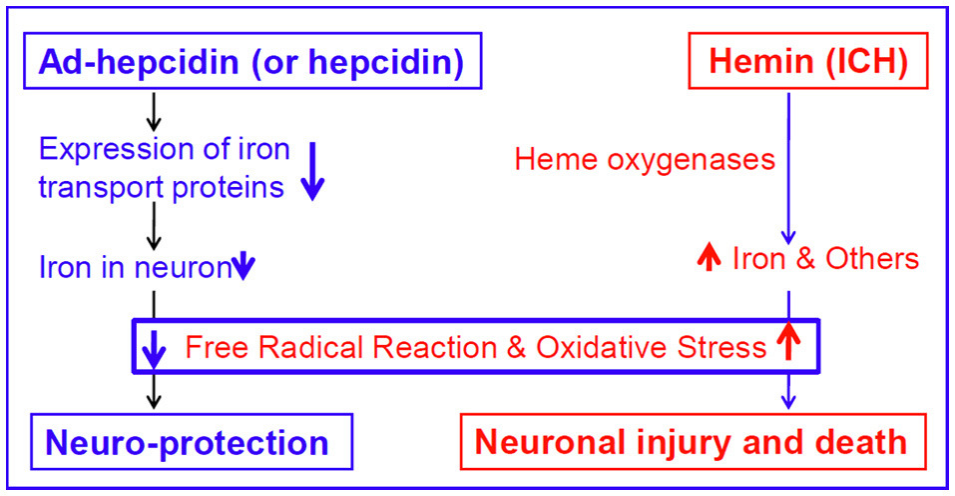
**The proposed mechanisms by which hepcidin protects neurons from hemin-induced injury**.

It has been well-demonstrated that iron is a major generator of reactive oxygen species (ROS), which are able to trigger a cascade of deleterious events that lead to cell damage or death (Halliwell, 1987; Qian et al., 1996; Dixon and Stockwell, 2014). Also, hemin itself can participate in redox reactions, producing free radicals that can damage intracellular structures and cause oxidative stress (Huffman et al., 2000). In addition, ROS have been domenstrated to be critical to the oxidative brain damage that occurs after ICH (Wang and Dore, 2007). Therefore, it should be reasonable to believe that hepcidin significantly reduce not only iron contents as we demonstrated, but also inhibit production of iron-mediated free radicals and oxidative stress in hemin-treated neurons, although we did not measure any indicators of ROS in the present study. In a recent study (Gong et al., 2016), we investigated the effects of pre-treatment of rats with ad-hepcidin on iron contents and iron-mediated free radical reaction and demonstrated that ad-hepcidin could prevent the increase in iron content as well as the level of dichlorofluorescein and 8-isoprostane, two reliable indicators of ROS in different brain regions of iron-overload rats. This finding plus the data of the present study imply that ad-hepcidin (and hepcidin) protect neuron from hemin-mediated injury firstly by reducing expression of iron transport proteins and then iron contents, secondly inducing a reduction in iron-mediated free radical reaction, and finally inhibiting oxidative stress-mediated damage (Figure 6).

Degradation of hemin by heme oxygenases produces not only iron, but also biliverdin and carbon monoxide (Kutty and Maines, 1981; Robinson et al., 2009). The findings from *in vitro* studies have suggested that neurons are particularly vulnerable to carbon monoxide and bilirubin (Miro et al., 1998; Silva et al., 2002; Brito et al., 2008) and demonstrated that bilirubin induces inflammation and oxidative stress (Fernandes et al., 2007; Brito et al., 2008). These results imply that carbon monoxide and bilirubin, the same as iron, may also have detrimental effects on brain cells in ICH although the detailed roles of these two breakdown products of hemoglobin in mediating secondary neuronal injury after ICH remain largely undefined. Currently it is unknown whether hepcidin has any roles on detrimental effects of carbon monoxide and bilirubin on brain cells in hemin-treated neurons. This issue needs to be explored in order to fully understand the mechanisms by which hepcidin protect neurons from hemin-induced injury.

## Author contributions

Design of work: YK and ZQ. Performed experiments: YZ, CZ, GY, MZ, JM, FZ. Data Analysis: YZ, CZ, GY, YK, and ZQ. Write paper: YK and ZQ.

### Conflict of interest statement

The authors declare that the research was conducted in the absence of any commercial or financial relationships that could be construed as a potential conflict of interest.

## Abbreviations

ICH: Intracerebral hemorrhage
Fpn1: Ferroportin 1
Ft-H: Ferritin heavy chain
Ft-L: Ferritin light chain
GFAP: Glial fibrillary acidic protein
LDH: Lactate dehydrogenase
MAP2: Microtubule associated protein 2
ROS: Reactive oxygen species
TfR1: Transferrin receptor1.
